# Species-specific regulation of PXR/CAR/ER-target genes in the mouse and rat liver elicited by *o*, *p'*-DDT

**DOI:** 10.1186/1471-2164-9-487

**Published:** 2008-10-16

**Authors:** Naoki Kiyosawa, Joshua C Kwekel, Lyle D Burgoon, Edward Dere, Kurt J Williams, Colleen Tashiro, Brock Chittim, Timothy R Zacharewski

**Affiliations:** 1 Department of Biochemistry and Molecular Biology, Michigan State University, East Lansing, Michigan, USA; 2 Center for Integrative Toxicology and the National Food Safety and Toxicology Center, Michigan State University, East Lansing, Michigan, USA; 3 Department of Pathobiology and Diagnostic Investigation, Michigan State University, East Lansing, Michigan, USA; 4 Medicinal Safety Research Laboratories, Daiichi Sankyo Co., Ltd. Fukuroi, Shizuoka, Japan; 5 Wellington Laboratories Inc. Guelph, Ontario, Canada

## Abstract

**Background:**

Dichlorodiphenyltrichloroethane (DDT) is a persistent estrogenic organochlorine pesticide that is a rodent hepatic tumor promoter, with inconclusive carcinogenicity in humans. We have previously reported that *o*, *p*'-DDT elicits primarily PXR/CAR-mediated activity, rather than ER-mediated hepatic responses, and suggested that CAR-mediated effects, as opposed to ER-mediated effects, may be more important in tumor promotion in the rat liver. To further characterize species-specific hepatic responses, gene expression analysis, with complementary histopathology and tissue level analyses were investigated in immature, ovariectomized C57BL/6 mice treated with 300 mg/kg *o*, *p*'-DDT, and compared to Sprague-Dawley rat data.

**Results:**

Rats and mice exhibited negligible histopathology with rapid *o*, *p*'-DDT metabolism. Gene expression profiles were also similar, exhibiting PXR/CAR regulation with the characteristic induction of *Cyp2b10 *and *Cyp3a11*. However, PXR-specific target genes such as *Apoa4 *or *Insig2 *exhibited more pronounced induction compared to CAR-specific genes in the mouse. In addition, mouse *Car *mRNA levels decreased, possibly contributing to the preferential activation of mouse PXR. ER-regulated genes *Cyp17a1 *and *Cyp7b1 *were also induced, suggesting *o*, *p*'-DDT also elicits ER-mediated gene expression in the mouse, while ER-mediated effects were negligible in the rat, possibly due to the inhibitory effects of CAR on ER activities. In addition, *o*, *p*'-DDT induced *Gadd45a*, *Gadd45b *and *Cdkn1*, suggesting DNA damage may be an additional risk factor. Furthermore, elevated blood DHEA-S levels at 12 h after treatment in the mouse may also contribute to the endocrine-related effects of *o*, *p*'-DDT.

**Conclusion:**

Although DDT is known to cause rodent hepatic tumors, the marked species differences in PXR/CAR structure, expression patterns and ligand preference as well as significant species-specific differences in steroidogenesis, especially CYP17A1 expression and activity, confound the extrapolation of these results to humans. Nevertheless, the identification of potential modes of action as well as species-specific responses may assist in the selection and further development of more appropriate models for assessing the toxicity of DDT to humans and wildlife.

## Background

Dichlorodiphenyltrichloroethane (DDT) is a persistent organochlorine pesticide that is a hepatic tumor promoter in the mouse and rat [1], with inconclusive carcinogenicity in the human liver [2,3]. Technical grade DDT is a mixture of *p,p'*-DDT, *o*, *p'*-DDT and their metabolites such as 1,1-dichloro-2,2-bis(p-chlorophenyl) ethylene (DDE) and l,l-dichloro-2,2-bis(p,p-chlorophenylethane) (DDD) [4]. *o*, *p'*-DDT exerts estrogenic effects in responsive tissues such as the uterus [5] by binding to estrogen receptor (ER)α and β subtypes [6]. In addition, *o*, *p*'-DDT is an agonist for pregnane X receptor (PXR) and constitutive androstane receptor (CAR) [7,8]. Thus, it may elicit complex responses through multiple nuclear receptors.

Several structurally diverse estrogenic compounds elicit ER-mediated responses in the liver [9,10]. We have previously reported that *o*, *p'*-DDT elicits primarily PXR/CAR-mediated, rather than ER-mediated hepatic responses, and suggested that CAR-mediated effects, as opposed to ER-mediated effects, may be more important in DDT-induced tumor promotion in the rat liver [11].

Although the rat is a preferred toxicological model because of the accumulated background knowledge regarding chemical-induced toxicity compared to mice, several studies report significant species-specific differences in response to chemical exposures. For example, 1,4-bis-[2-(3,5,-dichloropyridyloxy)] benzene (TCPOBOP) acts as potent phenobarbital-type enzyme inducer in the mouse liver but not in the rat or human. This is due to a substitution of Thr350 in mice with Met in the rat and human CAR [12-14]. Alternatively, another phenobarbital-type enzyme inducer 2,4,6-triphenyldioxane-1,3 induces hepatic CYP2B in rats but not in mice [15]. Such differences may affect CAR-mediated hepatic drug metabolism and disposition following xenobiotic exposure. In addition, rat genome annotation lags behind the mouse and human in maturity, confounding a more comprehensive assessment of rat gene expression data. Furthermore, targeted gene-disruption can be used in mice to further elucidate the roles of PXR, CAR and ER in *o*, *p*'-DDT elicited effects. Thus, we were motivated to investigate hepatic gene expression in the mouse following *o*, *p'*-DDT treatment to identify species-specific and conserved responses. Overall, the hepatic gene expression profiles were comparable, however there were marked species-specific differences in genes regulated by ER, which may involve species-difference in CAR activation. Furthermore, blood levels of DHEA-S, a precursor of androgen and estrogen, exhibited mouse-specific elevation, which may also contribute to endocrine system disruption. Thus, the present study further elucidates the dynamics of *o*, *p*'-DDT elicited responses in the rodent, and identifies species-specific responses that may be important to *o*, *p*'-DDT exposure for humans and wildlife.

## Methods

### Husbandry

Female C57BL/6 mice, ovariectomized on postnatal day 20 were obtained from Charles River Laboratories (Raleigh, NC) on day 25. Mice were housed in polycarbonate cages containing cellulose fiber chip bedding (Aspen Chip Laboratory Bedding, Northeastern Products, Warrensberg, NY) and maintained at 40–60% humidity and 23°C in a room with a 12 h dark/light cycle (7 am-7 pm). Animals were allowed free access to de-ionized water and Harlan Teklad 22/5 Rodent Diet 8640 (Madison, WI), and acclimatized for 4 days prior to dosing.

### Treatments and Necropsy

Mice (n = 5) were orally gavaged once or once daily for three consecutive days with 300 mg/kg b.w. *o*, *p'*-DDT (99.2% purity, Sigma-Aldrich, St Louis, MO) in 0.1 ml of sesame oil (Sigma-Aldrich) vehicle. An equal number of time-matched vehicle control animals (n = 5) were also treated in the same manner. Mice receiving one dose were sacrificed 2, 4, 8, 12, 18, and 24 h after treatment. Mice receiving three daily doses were sacrificed 24 h after the third treatment (72 h). All procedures were performed with the approval of the Michigan State University All-University Committee on Animal Use and Care. Animals were sacrificed by cervical dislocation and animal body weights were recorded. Whole liver weights were recorded and sections of the left lateral lobe (approximately 0.1 g) were snap-frozen in liquid nitrogen and stored at -80°C. The right lateral lobe was placed in 10% neutral buffered formalin (NBF, VWR International, West Chester, PA) for histopathology and stored at room temperature.

### Histopathology

Following fixation of the right lateral lobe for at least 24 h in 10% NBF, the samples were embedded in paraffin according to standard techniques. Five μm sections were mounted on glass slides and stained with hematoxylin and eosin. All embedding, mounting and staining of tissues were performed at the Histology Laboratory, (Department of Physiology, Michigan State University). The histopathology of each liver section was scored according to the NTP Pathology guidelines.

### Measurement of blood DHEA-S and androstenedione

Rat plasma samples were obtained from a previous study [11]. Mouse serum and rat plasma samples were used for determining dehydroepiandrosterone sulfate (DHEA-S) and androstenedione levels using a DHEA-S ELISA kit (Calbiotech Inc., Spring Valley, CA) and Androstenedione ELISA kit (Genway Biotech Inc., San Diego, CA), respectively. The detection limits, defined by the vendors, are 0.02 μg/ml of DHEA-S and 0.043 ng/ml of androstenedione, respectively.

### Protein preparation and Western analysis

Total protein was extracted from liver samples from all the time points using the Total Protein Extraction kit (Chemicon International Inc., Temecula, CA). Protein concentrations were determined using Bicinchoninic Acid Protein Assay Kit (Sigma-Aldrich). Total protein (100 μg) was resolved on a 11% denaturing SDS-polyacrylamide gel, and transferred to Hybond-ECL membrane (GE Healthcare, Waukesha, WI). Blots were incubated with blocking buffer (0.1% Tris-buffered saline pH 7.4 containing 1% low-fat dry milk) for 10 min at room temperature. Goat anti-CYP17A1, goat anti-actin antibodies and donkey horseradish peroxidase-conjugated anti-goat IgG were purchased from Santa Cruz Biotechnology Inc. (Santa Cruz, CA). Immunochemical staining was performed as described previously [16], with dilution of 1:500 (anti-CYP17A1), 1:500 (anti-actin) and 1:10000 (anti-goat IgG) using blocking buffer, respectively. SuperSignal West Dura substrate (Thermo Fisher Scientific, Inc., Waltham, MA) was used for signal detection. The Western analysis was performed on three independent biological replicates.

### RNA isolation

Total RNA was isolated from left lateral liver sections using TRIZOL Reagent (Invitrogen, Carlsbad, CA) and resuspended in The RNA Storage Solution (Ambion, Austin, TX). RNA concentrations were determined by spectrophotometry (A_260_) and purity was assessed by the A_260_:A_280 _ratio and by visual inspection of 3 μg on a denaturing gel.

### Microarray analysis

Temporal changes in gene expression were assessed using an independent reference design in which *o*, *p'*-DDT-treated samples were co-hybridized with time-matched vehicle controls using 3 biological replicates and 2 independent labelings of each sample (i.e. dye swap) for each time point. Whole Mouse Genome 4 × 44 K Oligo Microarray Kit (Agilent Technologies, Inc, Santa Clara, CA) was used for global gene expression analysis. All the reagents and enzymes were provided by Agilent Technologies, and the microarray analysis was performed according to the vendor's protocol. The microarrays were scanned at 635 nm (Cy5) and 532 nm (Cy3) using a GenePix 4000B microarray scanner (Molecular Devices, Union City, CA). Images were analyzed for feature and background intensities using GenePix Pro 6.0 (Molecular Devices). All data were managed in the toxicogenomic information management system dbZach relational database [17].

### Microarray data normalization and statistical analysis

Data were normalized using a semi-parametric approach [18]. Model-based *t*-values were calculated from normalized data, comparing treated and vehicle responses per time-point. Empirical Bayes analysis was used to calculate posterior probabilities (p1 [*t*]-value) of activity on a per gene and time-point basis using the model-based *t*-value [19]. Genes were filtered for activity based on the p1(*t*)-value. p1(*t*) values approaching one indicate changes in gene expression which are more robust. In this study, unique genes with a p1(*t*) > 0.999 for a minimum of two time points and absolute value fold change ≥ 1.5-fold compared to time-matched vehicle control for at least one time point were considered differentially expressed.

### Hierarchical clustering

A total of 996 orthologous rat and mouse genes were in common on the rat cDNA, mouse cDNA, and Agilent mouse oligonucleotide microarrays based on HomoloGene . Differentially expressed orthologs (|fold change| ≥ 1.5 for at least one time point in either species) for ethynylestradiol (EE)-treated mouse liver, *o*, *p'*-DDT treated rat liver and *o*, *p'*-DDT-treated mouse liver, were hierarchical clustered using Cluster 3.0 and TreeView software . In addition, differentially expressed genes known to be regulated by PXR, CAR and ER agonists [20-23], were identified from the literature and their expression profiles were subjected to hierarchical clustering.

### Correlation analysis

Correlation analysis was performed using relaxed criteria to allow for more inclusive comparison between mouse and rat data sets. Genes with a p1(*t*) > 0.99 and absolute fold change ≥ 1.5-fold at one or more time points in the *o*, *p'*-DDT-treated mouse liver samples were selected and used for correlation analysis. This analysis involved a multivariate correlation-based visualization application [24] that has been used to compare two independent gene expression profile datasets [11,25]. It calculates the temporal correlations between gene expression (fold change) and significance values (p1 [*t*]-value) for orthologous *o*, *p'*-DDT-treated mouse (this study) and *o*, *p*'-DDT-treated rat genes [11], and summarizes the results in a scatter plot.

### Quantitative Real-Time PCR (QRT-PCR)

Expression levels of mouse *Car *(*Nr1i3*), *Cyp17a1*, *Cyp2b10*, *Cyp3a11*, *Cyp7b1*, *Gadd45a*, *Gadd45b*, *Gapdh*, *Gclm*, *Pxr *(*Nr1i2*), and *Srebf1*, and rat *Gapdh *and *Cyp7b1 *were measured by QRT-PCR. For each sample, 2 μg of total RNA was reverse transcribed by SuperScript II using an anchored oligo-dT primer as described by the manufacturer (Invitrogen). The resultant cDNA (1.0 μl) was used as the template in a 30 μl PCR reaction containing 0.1 μM each of forward and reverse gene-specific primers designed using Primer3 [26], 3 mM MgCl_2_, 1.0 mM dNTPs, 0.025 IU AmpliTaq Gold and 1 × SYBR Green PCR buffer (Applied Biosystems, Foster City, CA). PCR amplification was conducted in MicroAmp Optical 96-well reaction plates (Applied Biosystems) on an Applied Biosystems PRISM 7000 Sequence Detection System using the following conditions: initial denaturation and enzyme activation for 10 min at 95°C, followed by 40 cycles of 95°C for 15 s and 60°C for 1 min. A dissociation protocol was performed to assess the specificity of the primers and the uniformity of the PCR generated products. Each plate contained duplicate standards of purified PCR products of known template concentration covering eight orders of magnitude to interpolate relative template concentrations of the samples from the standard curves of log copy number versus threshold cycle. The copy number of each unknown sample for each gene was standardized to that of *Gapdh *gene to control for differences in RNA loading, quality and cDNA synthesis. Primer sequences and amplicon sizes are provided as Additional file 1.

### Statistical analysis

Body weight, relative liver weight, hepatic concentrations of *o*, *p'*-DDT, *o*, *p'*-DDD, and *o*, *p*'-DDE and QRT-PCR data are presented as the mean ± SE. Statistical analysis was performed with two-way ANOVA followed by pairwise comparisons using Tukey's Honestly Significant Difference *post hoc *test to control Type I error (α = 0.05). For QRT-PCR data, the relative expression levels of target genes were scaled such that the standardized expression level of the time-matched vehicle control group was equal to 1 for graphing purposes. All statistics were performed using SAS 9.1.3 software (SAS Institute Inc., Cary, NC).

## Results

### Body Weight, Relative Liver Weight, and Histopathology

**Figure 1:**
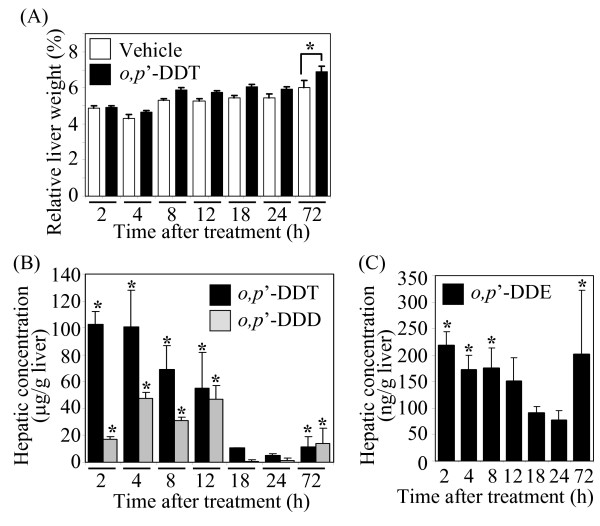
**Relative liver weight and tissue level analysis**. (A) Relative liver weight. Immature ovariectomized C57BL/6 mice were orally administered 300 mg/kg *o*, *p'*-DDT or sesame oil vehicle at time 0, 24 and 48 h. Mice were sacrificed 2, 4, 8, 12, 18, 24 or 72 h after the initial dose. The relative liver weight was significantly increased at 72 h. (B) Hepatic concentration of *o*, *p*'-DDT and *o*, *p*'-DDD. (C) Hepatic concentration of *o*, *p*'-DDE. Hepatic tissue levels of *o*, *p*'-DDT, *o*, *p*'-DDD and *o*, *p*'-DDE were determined using high-resolution gas chromatograph/HRMS from three randomly selected mice orally gavaged with 300 mg/kg *o*, *p*'-DDT. The data are presented as mean ± SE. The asterisk (*) indicates a significant (*p *< 0.05) difference from the vehicle controls.

Relative liver weight was significantly (*p *< 0.05) increased at 72 h compared to vehicle-treated mice (Fig. 1A). No effect on body weight was observed. These results are similar to those previously observed in the rat *o*, *p'*-DDT study [11]. There were no significant histological changes by *o*, *p*'-DDT treatment, except for two animals exhibiting cell death of small numbers of hepatocytes at 4 h or 72 h, where the nuclei were shrunken and hyperchromatic.

### Hepatic o, p'-DDT, o, p'-DDD and o, p'-DDE concentration

Animals treated with *o*, *p'*-DDT showed significantly greater levels of *o*, *p'*-DDT, from 2 to 18 h compared to vehicle controls (Fig. 1B). The levels of the metabolites *o*, *p*'-DDD and *o*, *p*'-DDE were significantly greater than controls from 2 to 12 h, and at 72 h (Figs. 1B and 1C), indicating that *o*, *p'*-DDT is readily metabolized in the liver, and that much of the *o*, *p'*-DDT and its metabolites remain primarily distributed to the liver from 2 to 12 h, similar to the rat [11]. Note that there was a statistical outlier in the 72 h treated group, which lead to significant *o*, *p*'-DDE and *o*, *p*'-DDD levels, but was still included in the analysis as there was no technical or biological justification for its removal.

### Microarray Data

**Figure 2:**
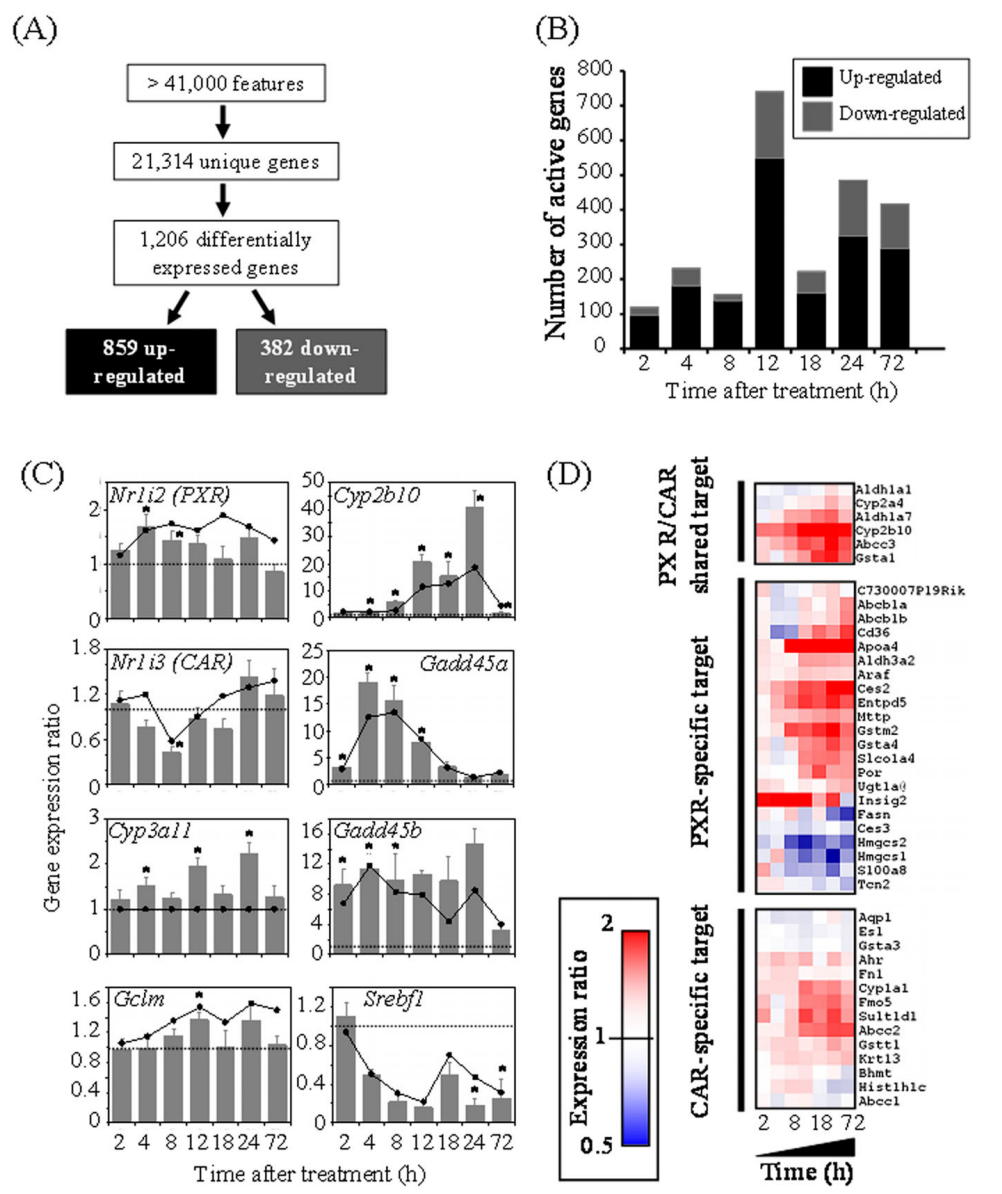
**Microarray and QRT-PCR results**. (A) Identification of differentially regulated genes. (B) Number of differentially expressed genes following *o*, *p'*-DDT treatment in the mouse liver. Differentially expressed genes were selected based on a p1(*t*) ≥ 0.999 at two or more time points and an absolute fold change ≥ 1.5 at one or more time points relative to time-matched vehicle controls. All differentially expressed genes are listed in Additional file 3. (C) Verification of microarray results by QRT-PCR. QRT-PCR results relative to time-matched vehicle controls are shown as bar and presented as mean ± SE. Microarray results are represented as lines. The dashed line indicates the expression level of the time-matched vehicle control. The asterisk (*) indicates a significant (*p *< 0.05) difference from the time-matched vehicle controls for QRT-PCR, n = 5. (D) Hepatic expression of PXR/CAR-target genes in *o*, *p'*-DDT-treated mouse. A heat map of *o*, *p'*-DDT elicited microarray expression profiles for selected PXR-, CAR-specific and PXR/CAR-shared target genes identified in the literature [20-23]. While some CAR-regulated genes such as *Cyp1a1*, *Fmo5*, *Sult1d1 *or *Abcc2 *were moderately induced, several PXR-target genes, including *ApoA4*, *Ces2*, *Gstm2 *or *Insig2*, exhibited strong induction. However, other PXR-target genes such as *Hmgcs1 *and *Hmgcs2 *were down-regulated.

Gene expression was assessed using the 4 × 44 K Mouse Agilent array containing approximately 44,000 oligonucleotide probes, representing 34,204 annotated genes including approximately 21,000 unique genes. Model-based *t*-values that compared treated and vehicle responses on a per time-point basis followed by Empirical Bayes analysis identified 1,206 differentially expressed genes across the time course based on a p1(*t*) > 0.999 for a minimum of two time points and absolute fold change ≥ 1.5-fold compared to time-matched vehicle control for at least one time point (Fig. 2A). Overall, 859 genes were induced while 382 genes were repressed. Changes in gene expression ranged from 36.4-fold induction for *Cyp2c55 *gene to -13.0-fold repression for gene *Car3*. The 12 h time point exhibited the greatest number of differentially expressed genes (Fig. 2B). All of the gene expression data as well as the identified differentially regulated genes are provided in Additional files 2 and 3, respectively.

A number of phase I drug metabolizing enzyme genes were induced such as *Cyp1a1 *(~2.2-fold), *Cyp2b9 *(~5.0-fold), *Cyp2b10 *(~18.4-fold), *Cyp2b13 *(~4.1-fold), *Cyp2c39 *(~2.1-fold), and *Cyp2c55 *(~36.4-fold) (Table 1). The induction of *Cyp2b10 *and *Cyp3a11 *were verified by QRT-PCR (Fig. 2C). The steroid metabolism genes, *Cyp17a1 *(~6.2-fold) and *Cyp7b1 *(~5.8-fold), were also induced, while *Cyp51 *and *Cyp7a1 *were down-regulated. Steroidogenic *Cyp11a1 *mRNA level was not detected by microarray or QRT-PCR (data not shown). Note that the hepatic induction of *Cyp17a1 *and *Cyp7b1 *by *o*, *p'*-DDT was observed in the mouse but not in the rat [11]. Several phase II genes (*Gsta1 *(~3.4-fold), *Gstm2 *(~3.2-fold), *Ugt2b35 *(~3.4-fold)) were also induced.

**Table 1:** Selected microarray results

Gene symbol	Entrez GeneID	Gene expression ratio (h)^a^						
		
		2	4	8	12	18	24	72
Drug metabolizing enzyme								
*Cyp1a1*	13076	**1.22**	**1.22**	1.22	**2.16**	1.78	1.92	1.42
*Cyp1a2*	13077	**1.16**	1.26	1.30	**1.61**	1.50	2.29	1.30
*Cyp2b9*	13094	**1.78**	**1.90**	2.41	**4.11**	**3.75**	**4.97**	**2.23**
*Cyp2b10*	13088	**2.19**	**2.12**	2.69	**11.37**	12.47	**18.42**	4.29
*Cyp2b13*	13089	**1.49**	**1.36**	1.46	**3.79**	3.54	**4.09**	2.04
*Cyp2c39*	13098	**1.25**	**1.41**	1.23	**1.51**	1.60	**2.13**	1.72
*Cyp2c55*	72082	**1.71**	**3.19**	7.43	**15.18**	30.38	**36.40**	**7.70**
								
Reductive reaction								
*Gsta1*	14857	1.30	**0.91**	1.39	**1.76**	3.00	**3.42**	2.37
*Gsta2*	14858	**1.53**	**0.83**	2.23	**3.52**	7.01	6.60	2.91
*Gstm2*	14863	1.03	**1.14**	2.20	**2.06**	2.44	3.19	1.90
*Gstt3*	103140	**1.22**	**1.17**	1.81	**2.31**	2.18	2.65	**1.62**
*Ugt2b35*	243085	**1.13**	**1.17**	1.94	**2.73**	2.30	**3.38**	**2.53**
								
DNA damage/apoptosis								
*Casp9*	12371	**1.01**	**0.67**	0.62	0.61	**0.64**	0.75	**0.83**
*Cdkn1a*	12575	**2.53**	15.36	**3.37**	**2.27**	**0.75**	1.54	**1.00**
*Fas*	14102	0.94	0.89	0.78	**0.80**	0.85	**0.66**	0.72
*Gadd45a*	13197	**2.96**	**12.58**	**13.54**	8.52	**3.16**	**1.41**	**2.29**
*Gadd45b*	17873	**4.55**	**8.35**	**6.66**	**7.83**	**5.12**	**6.80**	**3.32**
*Tnfrsf19*	29820	**1.22**	1.75	5.86	4.29	1.67	**1.66**	**2.21**
								
Metabolism								
*ApoA4*	11808	**1.15**	**1.05**	4.31	5.03	5.74	3.54	**3.34**
*Ces2*	234671	**1.16**	1.46	1.68	**2.22**	2.11	**3.36**	3.05
*Cyp17a1*	13074	**1.43**	**1.27**	3.97	**6.24**	5.52	**3.68**	**1.88**
*Cyp51*	13121	**0.98**	1.02	0.85	**0.39**	0.72	**0.35**	**0.49**
*Cyp7a1*	13122	1.07	0.58	0.25	0.44	0.66	0.51	**1.09**
*Cyp7b1*	13123	**0.98**	**1.51**	3.05	**5.77**	4.15	**5.44**	**3.41**
*Insig2*	72999	**2.80**	5.00	**4.40**	**4.63**	**1.45**	2.36	**0.81**
*Srebf1*	20787	0.94	0.51	0.31	0.21	0.70	**0.47**	**0.31**
								
Cell proliferation								
*Ccnb2*	12442	**0.94**	0.84	0.82	0.49	0.68	0.86	0.83
*Ccnd1*	12443	0.71	**1.17**	**1.08**	**0.85**	2.32	**0.78**	0.66
*Ccng2*	12452	1.26	**1.66**	1.39	0.71	**0.34**	1.03	0.95
*Mdm2*	17246	1.13	1.19	1.16	1.11	**0.97**	**1.09**	**1.28**
*Stmn1*	16765	**0.74**	1.00	0.69	0.35	0.59	0.84	0.60
								
Oxidative stress								
*Gclm*	14630	**1.06**	1.15	1.36	**1.54**	1.33	**1.59**	1.50
*Gsr*	14782	**1.02**	**1.40**	2.03	**2.47**	2.39	**2.54**	2.33
*Hmox2*	15369	**1.18**	**1.55**	1.44	**1.39**	**1.40**	**1.52**	**1.43**
*Nqo1*	18104	**0.99**	**1.10**	1.71	**1.97**	2.57	**2.35**	**2.43**
								
Transcription factor/signal transuction								
Ahr	*11622*	**1.20**	1.43	**1.29**	**1.50**	**0.96**	**1.47**	**1.15**
*Nr1i2 (PXR)*	18171	**1.16**	**1.63**	1.74	**1.62**	**1.90**	1.68	**1.44**
*Nr1i3 (CAR)*	12355	1.12	1.20	0.58	0.91	1.18	1.29	**1.38**

^a ^Highlighted values indicate expression ratio where p1(*t*) > 0.999.

A number of DNA damage-related genes such as *Cdkn1a *(~15.4-fold), *Gadd45a *(~13.5-fold), *Gadd45b *(~8.4-fold) and *Tnfrsf19 *(~5.9-fold) were induced following *o*, *p'*-DDT treatment, while *Fas *and *Casp9 *were down-regulated (~2.0-fold). The oxidative stress-responsive genes *Gclm *(~1.6-fold), *Gsr *(~2.5-fold), *Hmox2 *(~1.6-fold) and *Nqo1 *(~2.6-fold) exhibited induction. There was no clear induction for the cell proliferation-related genes such as *Ccnd1*, *Ccnb2*, *Mdm2 *or *Stmn1*, while their orthologs were induced by *o*, *p*'-DDT in the rat liver [11]. mRNA levels for *Ahr *and *Pxr *(*Nr1i2*) were slightly elevated, whereas *Car *(*Nr1i3*) mRNA levels decreased following *o*, *p'*-DDT treatment. *Srebf1 *which was previously shown to be down-regulated by *o*, *p*'-DDT in the rat liver [11], was also down-regulated in the mouse liver. Overall, the QRT-PCR results closely paralleled the expression pattern seen in the microarray data (Fig. 2C).

We also examined the expression of genes regulated by CAR, PXR or ER that were selected based on the null animal studies [20-23]. While CAR-specific targets such as *Cyp1a1 *(~2.2-fold), *Fmo5 *(~2.3-fold), *Sult1d1 *(~2.8-fold) and *Abcc2 *(~2.7-fold) showed relatively weak induction, several PXR-regulated genes were induced more strongly including *ApoA4 *(~5.8-fold), *Ces2 *(~3.4-fold), *Gstm2 *(~3.2-fold) or *Insig2 *(~5.0-fold) genes (Fig. 2D). However, other PXR genes, such as *Hmgcs1 *and *Hmgcs2*, were unexpectedly down-regulated.

### Comparative Analysis of Gene Expression

**Figure 3:**
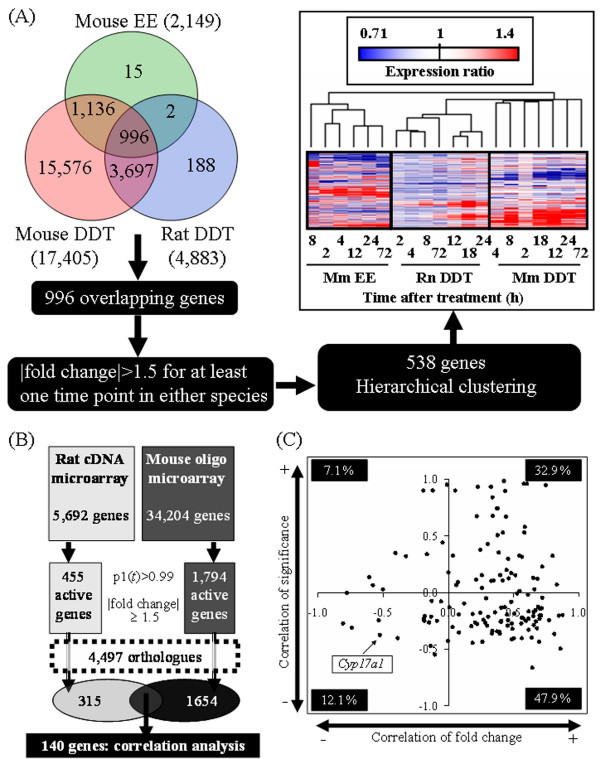
**Comparative analysis of global gene expression profiles elicited by *o*, *p*'-DDT**. (A). Comparative gene expression analysis between EE-treated mouse, *o*, *p'*-DDT-treated rat and *o*, *p'*-DDT-treated mouse. A total of 996 orthologs were represented on the rat cDNA microarray, mouse cDNA microarray and mouse Agilent oligonucleotide microarrays determined by HomoloGene . 538 of these orthologs showed a |fold change| ≥ 1.5 for at least one time point in either species. These 538 differentially expressed orthologs were subjected to hierarchical clustering. The dendrogram illustrates that mouse *o*, *p'*-DDT gene expression profiles are more similar to rat *o*, *p'*-DDT gene expression profiles than the mouse EE gene expression profiles. (B) Correlation analysis using differentially expressed orthologous genes. The temporal profiles of *o*, *p'*-DDT-treated mouse liver (current study) and those of the *o*, *p'*-DDT-treated rat liver [11] were compared by determining the Pearson's correlation of the temporal gene expression (fold change) and significance (p1 [*t*] value) between orthologs. Both studies used comparable study designs and data analysis methods, although different platforms were used (i.e., rat cDNA microarray and mouse Agilent oligonucleotide microarray). 140 genes were identified as differentially expressed orthologs. (C) Scatter plot of the 140 differentially expressed orthologous genes. Correlations for gene expression and significance approaching 1.0 indicate that the behavior or the orthologous genes are similar and would fall in the upper right quadrant. Orthologs tended to localize in upper- or lower-right quadrant (32.9% and 47.9% of total number of spots, respectively), indicating that temporal gene expression changes for *o*, *p'*-DDT-treated mouse and rat liver are comparable. However, poor correlations between the temporal p1(*t*) values and gene expression fold changes would fall within the lower left quadrant. For example, *Cyp17a1 *fell into this quadrant suggesting that significant differences exist between the rat and mouse ortholog expression profiles.

Hepatic gene expression profiles of EE-treated mice, *o*, *p'*-DDT-treated rats and *o*, *p'*-DDT-treated mice were compared by hierarchical clustering focusing on the 538 orthologs represented on both microarray platforms that satisfied the |fold change| ≥ 1.5 for at least one time point in any data set. The filtering criteria used here were relaxed compared to those used in Figs. 2A and 2B to include more orthologs in the correlation analysis, and thus be more informative of the overall similarity between the two data sets. Despite the differences in microarray platform (i.e., cDNA vs. oligonucleotide), *o*, *p'*-DDT elicited gene expression profiles in the rat and mouse that were more similar to each other than to the EE-treated mouse profile (Fig. 3A).

### Correlation Analysis

One hundred and forty orthologs were identified as differentially expressed on both rat cDNA and mouse oligo microarrays (Fig. 3B), which were further examined by correlation analysis to determine if their gene expression profiles were comparable. Again, the filtering criteria used here were relaxed compared to those used in Figs. 2A and 2B to include more orthologs in the correlation analysis. The scatter plot shows that a majority of the spots localized to either the upper-right (32.9%) or lower-right (47.9%) quadrants (Fig. 3C), indicating that the gene expression profiles between *o*, *p'*-DDT-treated rat and mouse were highly similar in terms of their gene expression pattern. Temporal correlation of the p1(*t*)-value (statistical significance) showed relatively low correlation between the two data sets, with 60% of spots located in the lower-right or lower-left quadrants. All correlation analyses results are provided in Additional file 4.

**Figure 4:**
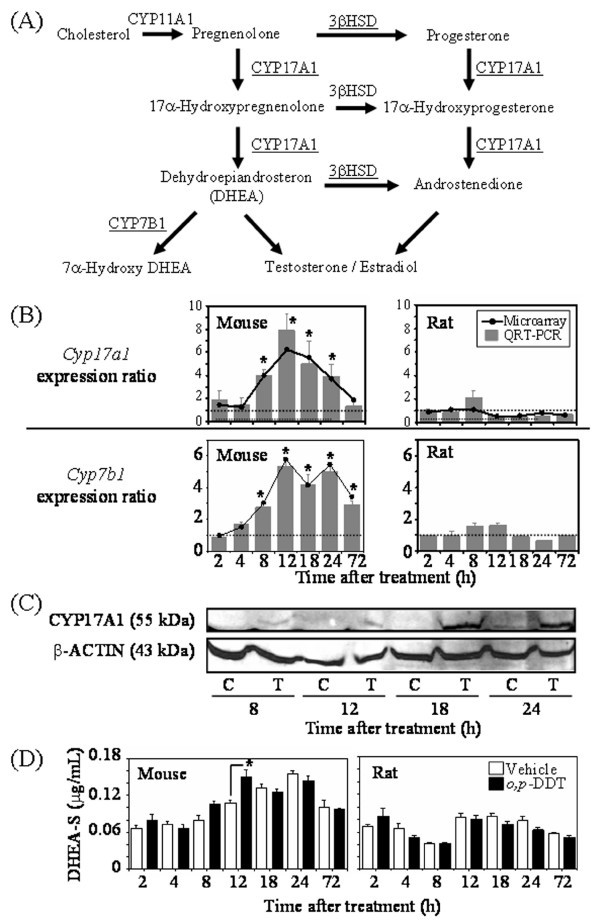
**Species-specific regulation of steroid hormone metabolism elicited by *o*, *p*'-DDT**. (A) Overview of the role of CYP17A1 and CYP7B1 in steroid metabolism. CYP17A1 metabolizes pregnenolone and progesterone to produce DHEA and androstenedione, respectively. Hepatic CYP7B1 is involved in bile acid biosynthesis, and also responsible for 7α-hydroxylation of DHEA. (B) Hepatic *Cyp17a1 *and (C) *Cyp7b1 *mRNA levels in the *o*, *p'*-DDT-treated mouse and rat. QRT-PCR results relative to time-matched vehicle controls are shown as bars and presented as mean ± SE. Microarray results are represented as lines. *o*, *p'*-DDT induced *Cyp17a1 *and *Cyp7b1 *mRNAs in the mouse liver, while it did not affect in the rat liver [11]. The dashed line indicates the expression level of the time-matched vehicle control. The asterisk (*) indicates a significant (*p *< 0.05) difference from the time-matched vehicle controls for QRT-PCR, n = 5. (C) Representative Western analysis result for hepatic CYP17A1 protein in *o*, *p'*-DDT-treated mouse liver. CYP17A1 protein levels were induced at 18 and 24 h. Western analyses were performed on 3 independent biological replicates to verify the consistency of the results. *C*, control; *T*, 300 mg/kg *o*, *p'*-DDT. (D) Blood DHEA-S levels. DHEA-S level was significantly higher at 12 h following *o*, *p'*-DDT treatment compared to time-matched controls in the mouse, while it did not change in rats.

For example, *Cyp17a1*, a key enzyme gene for steroidogenesis (Fig. 4A) fell into the lower left quadrant (Fig. 3C) suggesting divergent gene expression profiles in the mouse and rat. Fig. 4B clearly demonstrates that *Cyp17a1 *is significantly induced in the mouse and is non-responsive in the rat in both microarray and QRT-PCR data. *Cyp7b1*, another steroid metabolism gene, exhibited a similar species-specific gene expression profile (Fig 4B). Moreover, hepatic CYP17A1 protein induction was evident at 18 and 24 h in the mouse liver (Fig. 4C).

### Effects on DHEA-S Levels

Species-specific induction of CYP17A1 mRNA and protein levels suggests that steroid metabolism may also be differentially affected. More specifically, since CYP17A1 metabolizes pregnenolone and 17α-hydroxypregnenolone to produce DHEA (Fig. 4A), blood levels of DHEA-S were measured using an enzyme-linked immunosorbent assay. Serum DHEA-S levels exhibited a significant (*p *< 0.05) increase relative to vehicle at 12 h in the mouse, (Fig. 4D). In contrast, plasma DHEA-S was not significantly different between treated and control rats. Mouse serum androstenedione levels were below the detection level (data not shown).

## Discussion

*o*, *p'*-DDT elicits phenobarbital-type activities in the mouse liver, characterized by the increase in relative liver weight or induction of *Cyp2b10*, *Cyp3a11 *and *GST *mRNA levels [27,28]. In the rat, we previously reported that *o*, *p'*-DDT elicits PXR/CAR-mediated responses, with negligible ER-mediated activity [11], despite the responsive of the liver to estrogens [9,29], and the association of estrogenic activity with hepatic tumorigenesis [30]. Consequently, PXR/CAR-mediated responses to DDT may have a more significant role in hepatic tumor promotion [11]. In order to further investigate *o*, *p'*-DDT elicited gene expression mediated by PXR/CAR, we investigated its hepatic expression profile using a comparable mouse model, study design and data analysis [11]. Comparative analysis indicated that the hepatic gene expression profiles were similar between rat and mouse. However, correlation analysis of orthologous genes revealed that hepatic *Cyp17a1 *mRNA and protein levels as well as serum DHEA-S were only elevated in the mouse following *o*, *p'*-DDT treatment.

*Cyp17a1 *exhibits dose-dependent induction by EE in the mouse liver [10], suggesting ER-mediated induction. In addition, induction of *Cyp7b1 *and down-regulation of *Cyp7a1 *by EE are abolished in *ER alpha*-null mice but not in *ER beta-*, *Fxr*-, *Pxr*- or *Car*-null mice [23]. Collectively, these results indicate that *Cyp7a1 *mRNA levels are regulated by ERα in the mouse liver. Considering the strong inductions of *Cyp17a1 *and *Cyp7b1 *observed only in the mouse liver as well as down-regulation of *Cyp7a1 *in the mouse liver, *o*, *p'*-DDT may elicit ERα-mediated activity in the mouse liver but not in the rat liver.

In addition, the mouse profile suggests that *o*, *p'*-DDT elicited gene expression was predominately PXR regulated based on genes known to be regulated by PXR-, CAR- or PXR/CAR [20-23]. In part, this could be due to the repression of mouse *Car *mRNA levels by *o*, *p'*-DDT compared to its induction in the rat liver [11]. Furthermore, several genes associated with cell proliferation (*Ccnb1*, *Ccnb2, Mdm2 *and *Stmn1*) were induced in the rat liver [11], but not in the mouse. *Ccnd1*, a known CAR-regulated cell proliferation-related gene [31], was not significantly induced in the mouse, further supporting the hypothesis that *o*, *p'*-DDT preferentially activated PXR.

CAR also has an inhibitory effect on ER-mediated gene expression [32]. Therefore, the availability of CAR may inhibit ER-mediated effects elicited by *o*, *p'*-DDT in the rat liver [11], while the down regulation of *Car *mRNA in the mouse and the preferential activation of PXR may facilitate more ER-mediated gene expression. Consequently, ER-mediated responses may be important when assessing DDT-elicited hepatic responses in the mouse in addition to PXR/CAR-mediated response when compared to the rat.

CYP17A1 is an important enzyme in steroid biosynthesis that metabolizes pregnenolone and progesterone to produce DHEA and androstenedione, respectively [33,34]. Therefore, induction of CYP17A1 mRNA and protein levels may affect DHEA metabolism. *o*, *p*'-DDT treatment increased DHEA-S levels in the mouse whereas no change was detected in the rat. Although the liver is usually not a major steroidogenesis organ, microsomes are capable of participating in steroidogenesis [35]. In addition, hepatic microsomal CYP17A1 exhibits age-dependent expression, with higher expression and activity in immature rats [35,36]. *o*, *p'*-DDT elicited changes in other steroidogenic enzymes (e.g., *Cyp11a1) *and metabolites (e.g., androstenedione) were below the level of detection. Collectively, alterations in steroid levels following *o*, *p'*-DDT treatment could affect steroid levels, since peripheral tissues can use circulating DHEA and DHEA-S to produce androgens and estrogens [37]. Furthermore, the 7α-hydroxylation of DHEA and pregnenolone by *o*, *p'*-DDT induced CYP7B1 could also facilitate elimination of DHEA or the synthesis of neuro-active hormones [38,39].

In addition to PXR-, CAR- or ER-mediated gene expression changes, *o*, *p'*-DDT induced *Gadd45a*, *Gadd45b *and *Cdkn1*, all of which are DNA damage-responsive genes [40,41]. Consistent with this are the reports of the DNA damaging potential of DDT [42-44]. Consequently, DNA damage may be an additional risk factor for tumor initiation/promotion following *o*, *p'*-DDT exposure in addition to PXR/CAR- and ER-mediated activities. Considering that the induction of DNA damage-responsive genes precedes *Cyp2b10 *or *Cyp3a11 *induction, the DNA damage may not be caused by oxidative stress derived from enzyme induction. Moreover, *Gclm *and *Hmox*, both known oxidative stress-responsive genes [45,46], exhibited relatively weak induction compared to rats [11], suggesting that oxidative stress was not strongly induced.

## Conclusion

**Figure 5:**
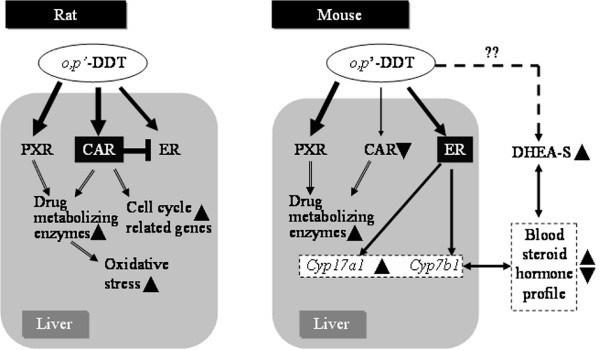
**Potential species-specific responses to *o*, *p'*-DDT**. In the rat liver, *o*, *p'*-DDT elicits PXR/CAR-mediated response, with negligible ER-mediated effects despite the established estrogenicity of *o*, *p*'-DDT in other target tissues (e.g., uterus). The absence of ER-mediated gene expression may be due, in part, to the inhibitory effects of CAR on ER. In contrast, *o*, *p*'-DDT elicits not only PXR/CAR-mediated responses but also ER-mediated effects such as the induction of *Cyp17a1 *and *Cyp7b1 *in the mouse liver. Gene expression profiling suggests that *o*, *p*'-DDT preferentially activates PXR in the mouse liver. Therefore, the inhibitory effects of CAR on ER-mediated signaling would be reduced in the mouse liver, allowing the activation of ER-mediated gene expression by *o*, *p*'-DDT. In addition, only mouse DHEA-S levels were increased, which could affect both endocrine and liver physiology. The induction of *Cyp17a1 *and *Cyp7b1 *may be involved in either the metabolism or disposition of DHEA, or the conversion of DHEA into other metabolites including neurosteroids.

In conclusion, *o*, *p'*-DDT elicits a broad spectrum of species-conserved and specific effects. This includes PXR/CAR- and ER-mediated responses, altered steroidogenesis, oxidative stress, and DNA damage (Fig. 5). Although DDT is known to cause liver tumors in both mice and rats, the marked species differences in PXR/CAR structure, expression patterns and ligand preference as well as significant species-specific differences in steroidogenesis, especially CYP17A1 expression and activity, confound the extrapolation of these results to humans. Nevertheless, the identification of potential modes of action as well as species-specific responses may assist in the development or selection of more appropriate models for assessing the toxicity of DDT to humans and wildlife.

## Abbreviations

CAR: constitutive androstane receptor (Nr1i3); Casp9: caspase 9; Ccn: cyclin; Cdkn1a: cyclin-dependent kinase inhibitor 1A; Cyp: cytochrome P450; DDT: dichlorodiphenyltrichloroethane; DHEA-S: dehydroepiandrosterone sulfate; EE: ethynylestradiol; ER: estrogen receptor; FXR: farnesoid X receptor; Gclm: glutamate-cysteine ligase modifier subunit; Gadd45: growth arrest and DNA-damage-inducible 45; Gsr: glutathione reductase; GST: glutathione *S*-transferase; Hmox2: heme oxygenase (decycling) 2; HMGCS: 3-hydroxy-3-methylglutaryl-Coenzyme A synthase; Hsd3b: hydroxy-delta-5-steroid dehydrogenase, 3 beta- and steroid delta-isomerase; Insig2: insulin induced gene 2; Mdm2: transformed mouse 3T3 cell double minute 2; Nqo1: NAD(P)H dehydrogenase, quinone 1; Stmn1: stathmin 1; PXR: pregnane X receptor (Nr1i2); QRT-PCR: Quantitative Real-Time PCR; Srebf1: sterol regulatory element binding factor 1; Stat: signal transducer and activator of transcription; TCPOBOP: 1,4-bis-[2-(3,5,-dichloropyridyloxy)] benzene; Tnfrsf19: tumor necrosis factor receptor superfamily member 19; UGT: UDP glycosyltransferase.

## Authors' contributions

NK performed all microarray, ELISA and Western assays, conducted and statistically analyzed QRT-PCR assays, as well as compiled and interpreted the data collected and generated the primary draft manuscript. JCK designed/conducted animal studies and participated in interpretation of microarray data. LDB provided database support for the microarray data including quality control assessments and statistical analysis. ED participated in microarray data analysis. KJW carried out histology examination. CT and BC carried out tissue level analysis. TRZ conceived the study and its design and supervised its completion. All authors read and approved the final draft of the manuscript.

## Supplementary Material

Additional File 1 **Primer sequence.** Click here for file

Additional File 2 ***o***, ***p'*****-DDT-treated mouse liver time-course study, full data set.** Blank gene expression ratios or p1(*t*) values are due to lack of or inapproprate statistical data for computation. Click here for file

Additional File 3 **Differentially regulated genes in the *o*, *p*'-DDT-treated mouse liver.**^a ^Highligted values indicate |fold change| > 1.5. ^b ^Highlighted values indicate p1(*t*) > 0.999. Click here for file

Additional File 4 **Correlation analysis between microarray results of *o*, *p*'-DDT-mouse and rat.** Click here for file
